# IPM reduces insecticide applications by 95% while maintaining or enhancing crop yields through wild pollinator conservation

**DOI:** 10.1073/pnas.2108429118

**Published:** 2021-10-25

**Authors:** Jacob R. Pecenka, Laura L. Ingwell, Rick E. Foster, Christian H. Krupke, Ian Kaplan

**Affiliations:** ^a^Department of Entomology, Purdue University, West Lafayette, IN 47907

**Keywords:** integrated pest management, neonicotinoid seed treatments, crop pollination, ecological intensification

## Abstract

Pest management practices in modern industrial agriculture have increasingly relied on insurance-based insecticides such as seed treatments that are poorly correlated with pest density or crop damage. This approach, combined with high invertebrate toxicity for newer products like neonicotinoids, makes it challenging to conserve beneficial insects and the services that they provide. We used a 4-y experiment using commercial-scale fields replicated across multiple sites in the midwestern United States to evaluate the consequences of adopting integrated pest management (IPM) using pest thresholds compared with standard conventional management (CM). To do so, we employed a systems approach that integrated coproduction of a regionally dominant row crop (corn) with a pollinator-dependent specialty crop (watermelon). Pest populations, pollination rates, crop yields, and system profitability were measured. Despite higher pest densities and/or damage in both crops, IPM-managed pests rarely reached economic thresholds, resulting in 95% lower insecticide use (97 versus 4 treatments in CM and IPM, respectively, across all sites, crops, and years). In IPM corn, the absence of a neonicotinoid seed treatment had no impact on yields, whereas IPM watermelon experienced a 129% increase in flower visitation rate by pollinators, resulting in 26% higher yields. The pollinator-enhancement effect under IPM management was mediated entirely by wild bees; foraging by managed honey bees was unaffected by treatments and, overall, did not correlate with crop yield. This proof-of-concept experiment mimicking on-farm practices illustrates that cropping systems in major agricultural commodities can be redesigned via IPM to exploit ecosystem services without compromising, and in some cases increasing, yields.

## Significance

Environmental damage from insecticide overuse is a major concern, particularly for conservation of “good” insects such as pollinators that ensure stable production of food crops like fruits and vegetables. However, insecticides are also necessary for farmers to manage “bad” insects (i.e., pests), and thus, a more holistic view of crop management needs to account for the proper balance between the beneficial and detrimental aspects of pesticides. Here, we used multiyear field experiments with a paired corn–watermelon cropping system to show that insecticide use can be dramatically reduced (by ∼95%) while maintaining or even increasing yields through the conservation of wild bees as crop pollinators. These data demonstrate that food production and ecosystem sustainability are not necessarily conflicting goals.

Integrated pest management (IPM) is a central organizing principle to guide pesticide use. At its core, IPM is designed to optimize pesticide inputs, preventing overuse via practices such as scouting with applications dictated by a range of parameters, including economic thresholds, heat unit accumulations, and historical data (i.e., a use-as-needed approach). Although IPM has been a mainstay in agriculture for >50 y (1), technological and philosophical changes in farming practices over recent decades have made this well-accepted and effective approach to pest management far more difficult to implement in practice (2, 3). A contributing factor to this trend is the introduction and widespread adoption of prophylactic neonicotinoid seed treatments (NSTs) on staple crops such as corn, soybean, cotton, and wheat (hereafter “row crops”). Unlike some transgenic crops (i.e., Bt hybrids), NSTs were not developed in response to new or recurring pest outbreaks; in fact, pest populations remain at historic lows in many US crops (4, 5). As a result, studies have struggled to document a clear agronomic or economic benefit from using NSTs in the United States and Canada (6–13), likely due to the sporadic occurrence of the pests they are purported to control. In a recent analysis, <5% of corn fields in Quebec experienced a measurable benefit from the use of NSTs (14). Yet, >90% of corn and >50% of soybean and cotton seed is coated with a neonicotinoid in the United States (15, 16). NSTs could, in theory, conform to an IPM framework if proactive, insurance-based pest management is justified by persistent pest pressures (17); however, the existing data largely do not support this view, especially in northern temperate regions (e.g., the US “Corn Belt”).

The lack of yield benefit from NSTs is also concerning due to accumulating evidence of nontarget effects from their overuse (18–20). When evaluated, <5% of NSTs were absorbed by the crop (21), with the remaining active ingredient lost to the greater ecosystem (10, 22), where it can persist for years in groundwater (23, 24) and soil (25, 26). The pervasive use of NSTs has led to contamination of waterways near crop fields (27), noncrop wild plants (28–30), pollen and nectar in honey bee colonies (31–33), and even human hair (34) and drinking water (35).

Although a wide diversity of nontarget animals is vulnerable to neonicotinoid exposure, pollinating insects have been the most well-studied group, in no small part because of global declines in bee populations (36, 37). The insecticidal toxic load for honey bees has dramatically increased over the past 20 to 30 y despite declining application volume (38, 39). This change was most evident in the US Heartland, with a 121-fold increase in oral toxicity, an effect attributed almost completely to corn and soybean NSTs. These patterns suggest that neonicotinoid inputs in row crops have the potential to profoundly affect pollinator health across landscapes, with potential reverberations in noncorn/soybean habitats.

Most fruits, vegetables, and tree nuts (hereafter “specialty crops”) are at least partially—and, in some cases, entirely—reliant on insect pollinators for yield (40–42). Consequently, NST-mediated impacts have the potential to threaten food production. However, the crops driving neonicotinoid exposure are not the same ones that depend on bees for their services. Corn, soybean, and cotton account for >80% of neonicotinoid use (15), but both soybean and cotton are primarily considered self-pollinating [despite some recent evidence for yield benefits with bee visitation (43, 44)], and corn is wind-pollinated. Although bees are known to visit these crops for nectar and/or pollen, insect pollinators are not critical to their production. Row crops are cultivated over a large fraction of arable land in the United States [9.8% of the continental United States is dedicated to corn, soybean, and cotton (45)], and specialty crop fields in this region are often adjacent to at least one of these row crops; therefore, we may expect carryover effects of NSTs on specialty crop pollination. For example, NST-infused dust from corn planting moves hundreds of meters beyond the field border (10, 32, 46), resulting in honey bee mortality (summarized in ref. 47). Thus, the relatively smaller areas devoted to specialty crops may invariably experience extrafield exposure from nearby row crops. Similarly, specialty and row crops are common rotation partners, resulting in neonicotinoid soil residues that impact ground-nesting bees (48–50). These spatial and temporal avenues generate several possible exposure routes. A simulation model (46) using field-derived values predicted that NSTs from corn planting in late spring erode honey bee population size enough to reduce capacity for blueberry and cranberry pollination later that summer, resulting in the potential for economic losses to neighboring berry growers. A similar outcome was demonstrated when modeling almond pollination potential for honey bee colonies that reside in the corn-dominated Northern Great Plains for much of the year (51).

In the work described here, we empirically test the hypothesis that IPM implementation, consisting of pest thresholds and removal of NSTs, dramatically reduces insecticide use and improves pollinator function without sacrificing crop yields. To do so, we used a multiyear, multisite field study, conducted in a dual cropping system representative of agriculture in the midwestern United States, and other parts of the world, consisting of a smaller acreage specialty crop paired with (i.e., adjacent to and grown in rotation with) a larger acreage row crop. We compared the effects of IPM versus conventional insecticide practices across several key metrics: insect pest abundance and damage, pollination, and yield. This design is unique in integrating field measurements of all factors across years, locations, and cropping systems. We paired field corn and seedless watermelon—a functionally dioecious crop that requires bees to move pollen between plants for fruit production. The experiment was conducted over 4 y (2017 to 2020) across five sites in Indiana, a state that is typically ranked in the top five nationally for both corn and watermelon production (52). In the conventional management (CM) system, we applied industry-standard practices used by growers in the region, characterized by NSTs on corn and preventative, calendar-based insecticides on watermelon. In the IPM system, we used NST-free corn seed with watermelon inputs determined by population thresholds established for arthropod pests. We predicted that the IPM system would have both higher pest densities (while remaining below economic thresholds) and pollinator visitation rates, resulting in equivalent (corn) or higher (watermelon) crop yield and increased farm profitability. This field experiment provides a comprehensive reassessment of IPM principles for both modern row crop and specialty crop pest management in the highly productive and intensively managed agricultural region of the midwestern United States.

## Results

### IPM Systems Experienced Infrequent Pest Outbreaks, Requiring Few Insecticide Inputs.

Neonicotinoid seed treatments target early-season pests; however, early-season corn damage was unaffected by NSTs with corn plant stand similar (*P =* 0.867) between IPM (11,040 ± 145 plants ⋅ ha^−1^) and CM (11,052 ± 106 plants ⋅ ha^−1^) fields (*SI Appendix*, Fig. S3; refer to *SI Appendix*, Table S6*A* for full statistical model for this and subsequent pest metrics). Similarly, during the first 3 y of the study, <1% of sampled plants showed any direct evidence of feeding by western corn rootworm *Diabrotica virgifera virgifera* LeConte—the primary insect pest of corn in this region—across both treatments (overall damage rating: 0.001 ± 0.000 nodes). In the fourth and final year (2020), damage was more prevalent, with 33% of IPM corn roots showing evidence of rootworm feeding. This pattern resulted in a significant treatment × year interaction (*P* = 0.006), with pairwise comparisons showing that IPM fields in 2020 had higher damage ratings than all other treatment × year combinations (*SI Appendix*, Fig. S4). Despite this statistical increase in pest pressure in the IPM treatment over time, the magnitude of the effect was low (2020 IPM damage rating (on a 0-to-3 scale): 0.17 ± 0.07 nodes).

**Fig. 1.**
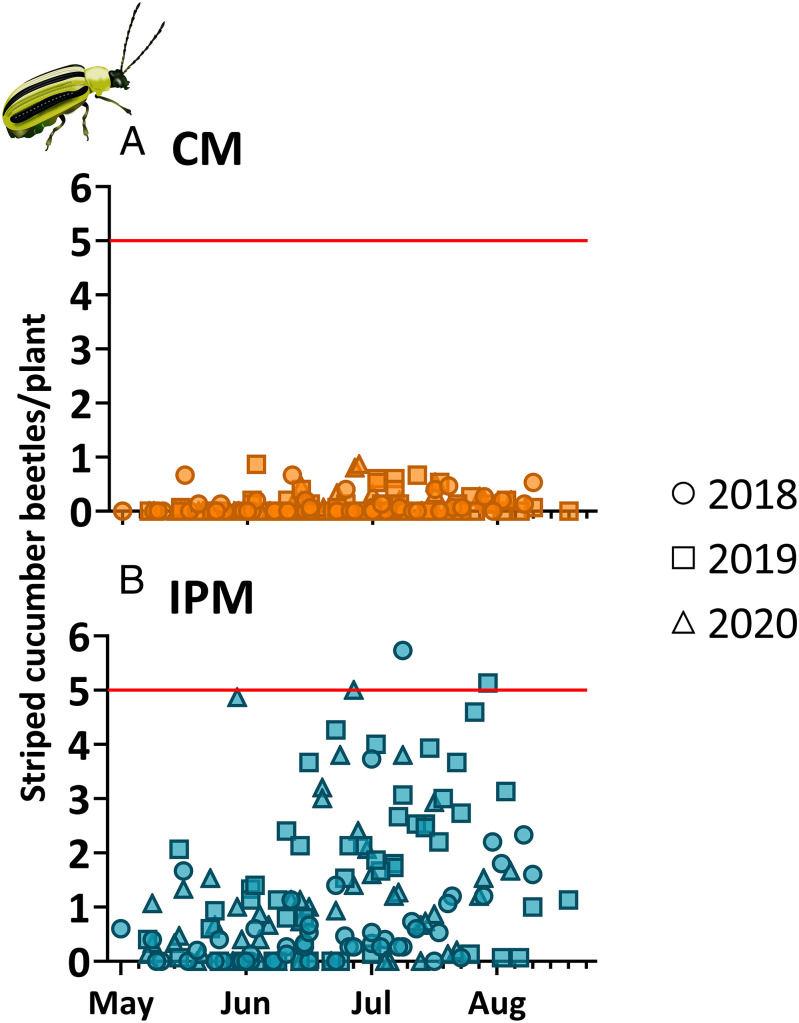
SCBs were higher in IPM watermelon fields, but infrequently reached levels associated with economic loss. Watermelon fields within both a CM (*A*) and IPM (*B*) system were scouted weekly, and each point represents a 15-plant average of SCBs from seedling transplant until fruit harvest. Red lines in each graph indicate the five-beetle/plant economic threshold, while circles (2018), squares (2019), and triangles (2020) differentiate experiment years. In IPM fields, in each instance in which beetle levels reached the economic threshold, insecticide was applied <2 d following the survey.

Watermelon in the CM treatment received insecticide sprays on a predetermined schedule that did not depend on scouting. These calendar applications maintained populations of the primary insect pest—striped cucumber beetle (SCB) *Acalymma vittatum* (F.)—well below the published economic threshold of five beetles per plant (Fig. 1*A*; seasonal mean SCBs per plant = 0.11 ± 0.05). In IPM fields, SCBs also rarely reached their economic threshold (Fig. 1*B*; seasonal mean SCBs per plant = 1.18 ± 0.34). Over the 3-y experiment, only four total IPM insecticide sprays (2018: 1; 2019: 1; and 2020; 2) were required across all five sites combined (i.e., four applications in 15 site-year growing seasons). In contrast, 77 insecticide applications were made in the CM treatment over the same period across all sites. In the IPM treatment, a single spray per field was sufficient to keep populations below economic thresholds for the remainder of the season; however, in most site-years, even a single spray was unnecessary. Appearance of secondary pests—primarily aphids and spider mites—occurred under both management systems (CM = 6, IPM = 4), but, interestingly, these populations only warranted additional pesticide applications (*n* = 2) in the CM plots (*SI Appendix*, Table S5). All other observed secondary pests did not spread to neighboring plants and were likely controlled by abiotic factors (heavy rain) or natural enemies, which were confirmed by the presence of parasitized aphids or coccinellid larvae/adults on flagged plants known to be previously infested.

### Pesticide Residues Were Higher in Conventionally Managed Systems.

Neonicotinoids applied to both crops in the CM system were routinely found in sampled plant tissues and soil; 99% (*n* = 335) of all samples collected had residues of at least one neonicotinoid compared to only 65% (*n* = 221) of IPM samples.

Neonicotinoids in the pollen of both crops were higher in the CM than IPM treatment. Watermelon pollen had consistently higher concentrations of imidacloprid in CM (median: 6.17 ng/g) compared to IPM (median: < limit of detection [LOD]) flowers (Table 1); however, residues in CM fields decreased over time, with highest values in early-blooming flowers (*SI Appendix*, Table S8). Both clothianidin (CM: 49%, IPM: 5%) and thiamethoxam (CM/IPM median: < LOD) were infrequently detected at low levels in watermelon flowers. Corn pollen, on the other hand, rarely contained imidacloprid residues (CM: 50%, IPM: 10%), but CM corn pollen contained higher levels of both clothianidin (93% detection, median: 1.91 ng/g) and thiamethoxam (100% detection, median: 2.01 ng/g) than IPM corn pollen, which only contained detectable amounts of clothianidin and thiamethoxam in 20% and 10% of all samples, respectively (Table 2). This low-level contamination is likely attributable to uptake of carryover NSTs from previous cropping seasons before the experiment began or from adjacent fields.

**Table 1.** Neonicotinoids were more frequently detected in watermelon pollen from fields under conventional management

	Neonicotinoid residue in watermelon pollen					
	Conventional			IPM		
Year	Percent detection (25)	Median (ng/g)	Range (ng/g)	Percent detection (25)	Median (ng/g)	Range (ng/g)
	*Imidacloprid*					
2018	96%	4.43	< LOD-82.53	0%	< LOD	< LOD
2019	100%	6.28	1.38 to 55.86	44%	< LOD	< LOD-1.69
2020	100%	4.84	1.54 to 22.94	4%	< LOD	<LOD-0.95
	*Clothianidin*					
2018	24%	< LOD	< LOD-2.12	0%	< LOD	< LOD
2019	72%	0.50	< LOD-1.15	0%	< LOD	< LOD
2020	52%	0.14	<LOD-0.79	0%	< LOD	< LOD
	*Thiamethoxam*					
2018	24%	< LOD	< LOD-0.21	0%	< LOD	< LOD
2019	16%	< LOD	< LOD-0.87	12%	< LOD	< LOD-0.16
2020	28%	< LOD	< LOD-0.25	8%	< LOD	< LOD-0.15

LC-MS/MS was used to quantify imidacloprid, clothianidin, and thiamethoxam from fields (*n* = 10). Watermelon represents pooled samples (3 g from 50 to 100 flowers) from each field across five consecutive weeks during peak bloom (n = 25 per year). LOD was 0.03, 0.01, and 0.025 ng/g for clothianidin, thiamethoxam, and imidacloprid, respectively.

**Table 2.** Neonicotinoids were more frequently detected in corn pollen from fields under conventional management

	Neonicotinoid residue in corn pollen					
	Conventional			IPM		
Year	Percent detection (10)	Median (ng/g)	Range (ng/g)	Percent detection (10)	Median (ng/g)	Range (ng/g)
	*Imidacloprid*					
2018	10%	< LOD	< LOD-0.11	0%	< LOD	< LOD
2019	30%	< LOD	< LOD-0.73	0%	< LOD	< LOD
2020	100%	0.23	0.11 to 0.69	30%	<LOD	<LOD-0.71
	*Clothianidin*					
2018	70%	2.00	< LOD-4.66	10%	< LOD	< LOD-0.85
2019	100%	1.94	0.42 to 4.54	10%	< LOD	< LOD-0.12
2020	100%	1.91	0.30 to 2.77	40%	< LOD	< LOD-0.24
	*Thiamethoxam*					
2018	100%	2.01	0.65 to 4.18	0%	< LOD	< LOD
2019	100%	2.50	0.94 to 2.98	0%	< LOD	< LOD
2020	100%	1.81	0.33 to 2.54	30%	< LOD	< LOD-0.56

LC-MS/MS was used to quantify imidacloprid, clothianidin, and thiamethoxam from fields (*n* = 10). Corn pollen was taken during anthesis with two replicates per field. LOD was 0.03, 0.01, and 0.025 ng/g for clothianidin, thiamethoxam, and imidacloprid, respectively.

Neonicotinoid residues were also higher in soil and leaf samples within the CM management system, depending on sample date. Refer to *SI Appendix*, Tables S7–S9 for pesticide summary data across all sample types and years. Nonneonicotinoid pesticides applied to the system—fungicides and the pyrethroid lambda-cyhalothrin—were also detectable but at varying levels (*SI Appendix*, Table S10). In general, fungicide detection was roughly equivalent across CM and IPM fields, whereas lambda-cyhalothrin was more frequently detected in watermelon leaves and pollen in CM fields (but overall detection rates were relatively low; <20% of samples).

### IPM Enhanced Watermelon Pollination.

The pollinator community composition was broadly similar across treatments, with the most commonly observed taxa being honey bees, *Apis mellifera* (CM = 35%, IPM = 13%), *Melissodes* sp. (CM = 22%, IPM = 25%), and *Lasioglossum* + *Halictus* sp. (CM = 26%, IPM = 37%) (refer to *SI Appendix*, Fig. S5 and Table S11 for a complete description across taxa). Overall abundance of pollinators visiting flowers was 99% greater in IPM (0.64 ± 0.05 pollinators ⋅ min^−1^) than CM (0.32 ± 0.02 pollinators ⋅ min^−1^) fields (refer to *SI Appendix*, Table S6*B* for full statistical model for this and subsequent pollination metrics). Notably, this pattern was driven entirely by wild bees. When treatment effects were tested for managed and wild species as separate groups, there was no impact on honey bee visitation (*P* = 0.202), but wild bee visitation was lower (*P* < 0.001) in CM fields.

**Fig. 2.**
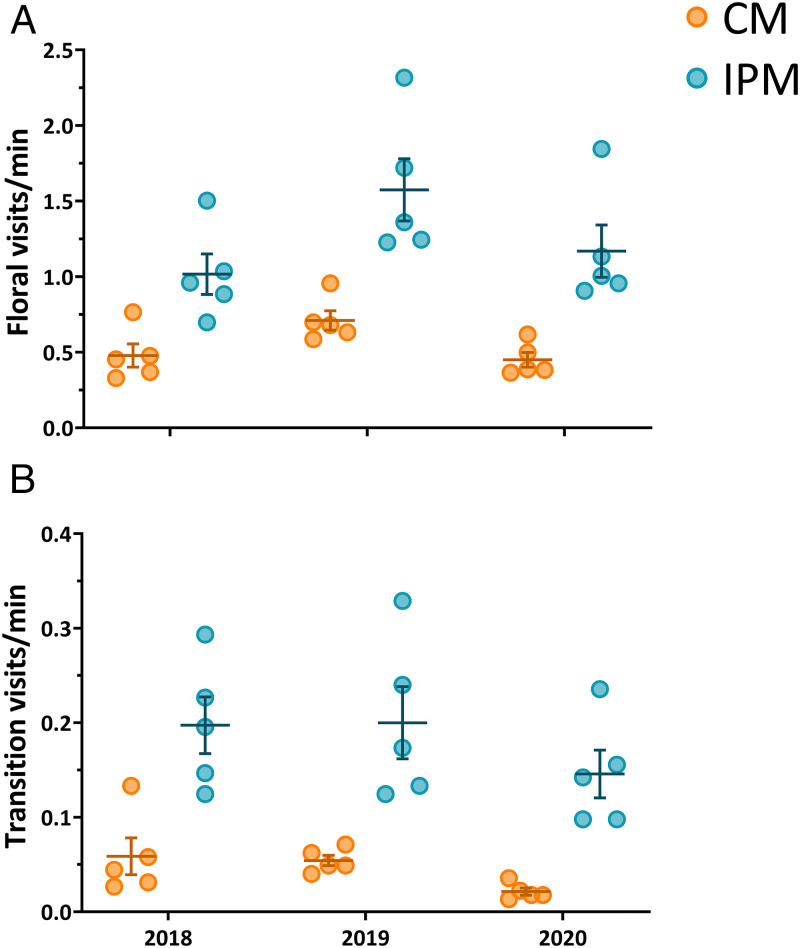
The rate of visits to watermelon flowers (*A*) and transition visits from a male to female flower (*B*) were both significantly higher in IPM fields. Each point within a cluster (*n* = 5) represents all observations from a single site during that field season (225 observation minutes). Whiskers within the plot show the mean ± SEM of all sites within each cluster.

Number of flowers visited per minute was 129% greater in IPM (1.25 ± 0.11 visits ⋅ min^−1^) than in CM (0.55 ± 0.05 visits ⋅ min^−1^) fields (Fig. 2*A*). Also, transition visits (observed trips from male to female flower) were 305% higher in IPM (0.18 ± 0.02 transition visits ⋅ min^−1^) than CM (0.05 ± 0.01 transition visits ⋅ min^−1^) fields (Fig. 2*B*).

### NSTs Did Not Affect Corn Yield.

**Fig. 3.**
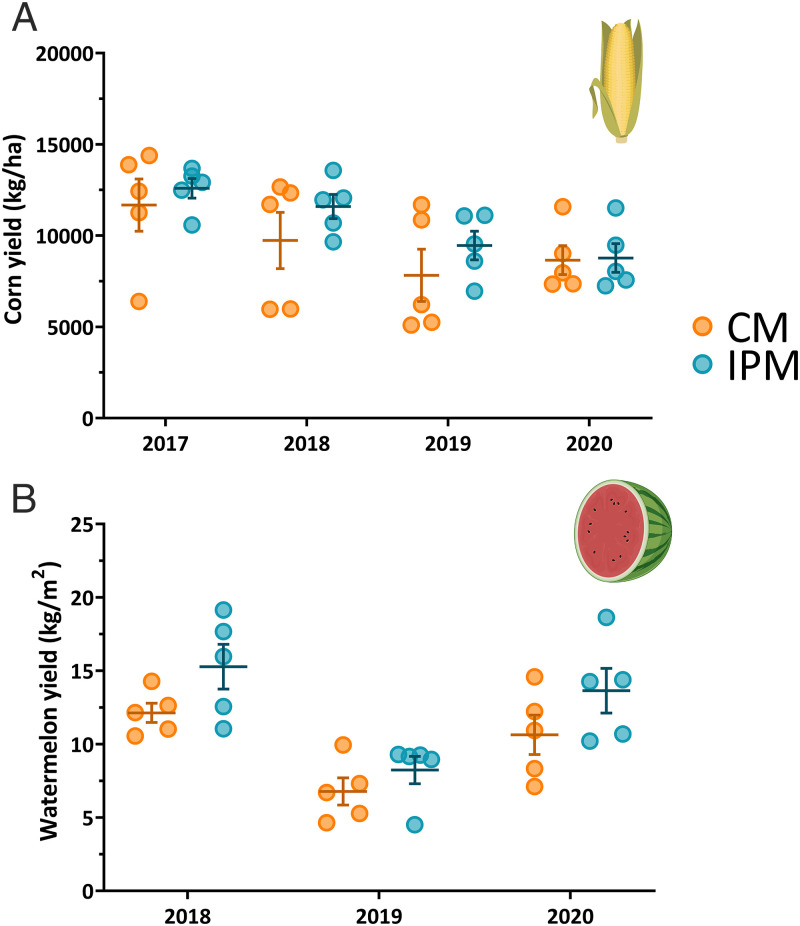
Corn yield was unaffected by CM system (*A*), but watermelon yield was significantly higher when grown under an IPM system (*B*). Each point within a cluster (*n* = 5) represents the yield from a site during that field season. Whiskers within the plot show the mean ± SEM of all sites within each cluster. Corn and watermelon icons from BioRender.

There was no statistical difference (*P =* 0.097) in corn yields between management systems, but there was a trend for higher yield in IPM (10,602 ± 479 kg/ha) compared to CM (9,471 ± 694 kg/ha) fields (Fig. 3*A*; refer to *SI Appendix*, Table S6*C* for full statistical model for this and subsequent yield metrics). Similarly, we conducted a more targeted small-plot trial in 2019 with higher replication and better control of local environmental factors. This follow-up experiment also showed no difference (F_1,51_ = 0.47, *P* = 0.501) between +NST (12,688 ± 269 kg/ha) and −NST (12,511 ± 311 kg/ha) corn yields (*SI Appendix*, Fig. S6).

### IPM Watermelons Produced Higher Yields by Preserving Wild Bees.

Watermelon yield was 25.7% higher in IPM (9.91 ± 0.84 kg/m^2^) than in CM (7.88 ± 0.63 kg/m^2^) fields (Fig. 3*B*). The significant difference in overall yield between treatments (*P* = 0.002) was driven by the reduced number of watermelons harvested in CM (59.07 ± 4.15) compared to IPM (72.13 ± 5.51) plots. Individual fruit weights were not statistically different (*P* = 0.071), but IPM melons (6.76 ± 0.18 kg) tended to be larger than those from CM (6.22 ± 0.23 kg) fields. Yield data only included fruit deemed marketable without any rind damage from insect feeding or other deformities. IPM watermelons experienced an increased number of damaged fruits (55 deemed unmarketable in IPM with only 1 in CM fields); this represented a <5% loss in potential yield.

**Fig. 4.**
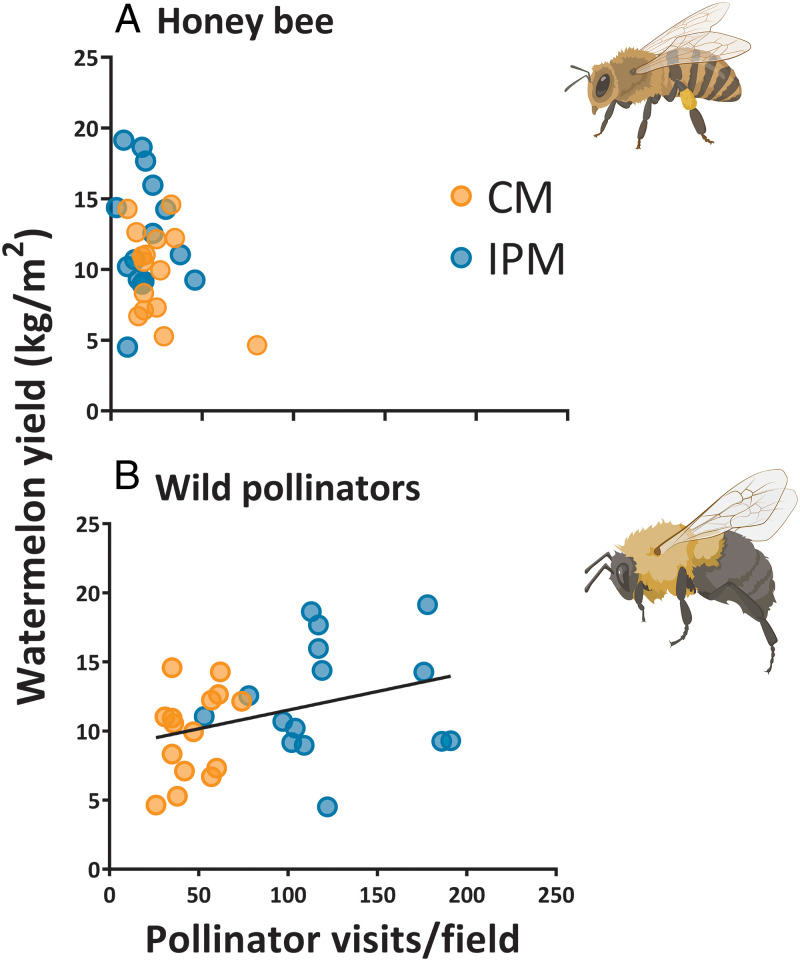
Honey bees (*A*) did not predict watermelon yield, but increased wild pollinator visitation (*B*) in the IPM fields resulted in higher watermelon yield. All plots were stocked with two honey bee colonies at opposite corners of the field. Each point is the total number of observed pollinator visits at a field per site (*n* = 5 sites with 225 observation minutes) and the corresponding site’s average watermelon yield. Best-fit trend line shows relationship using regression model with *P* < 0.05. Bee icons from BioRender.

There was no relationship between total pollinator visitation and crop yield, likely due to the high stocking of managed honey bee colonies in both pest management systems. To test this possibility, we separately analyzed honey bees apart from the wild bee community. This subset analysis confirmed that honey bee visitation could not predict watermelon yield (Fig. 4*A*; overall slope, *P* = 0.097), whereas higher rates of wild pollinator visitation, driven by lower insecticide use, resulted in correspondingly higher watermelon yield (Fig. 4*B*; overall slope, *P* = 0.043; CM slope, *P* = 0.218; IPM slope, *P* = 0.728).

### IPM Was More Profitable than Conventional Management.

The product cost (i.e., no application cost) of Cruiser 5FS on corn was $31.10 ⋅ ha^−1^; however, using industry-provided data (53), the inflation-adjusted cost of an NST at the rate applied in this study was $57.79 ⋅ ha^−1^. Using this cost calculation and the range of field sizes, the use of an NST in CM corn represented a cost of $330.93 ± 30.93 ⋅ field^−1^. The cost relative yield (CRY; the minimum percentage in yield gain in which the insecticide cost is recuperated) was 3.3%, which was not reached in either the CM/IPM experiment or the within-site NST evaluation, indicating that the cost of NST was not recovered at any of the sites in this experiment.

Watermelon insecticides in the CM system cost $44.05 ⋅ ha^−1^ for the soil drench and $50.28 ⋅ ha^−1^ for all foliar insecticide applications ($12.57 per application) for a total cost of $94.33 ⋅ ha^−1^ on each field with additional applications required to control secondary pests in some fields, increasing this cost. While several insecticide sprays were applied to the IPM watermelons, this was a minority of fields, leading to an average cost for IPM insecticides at $3.35 ± 1.44 ⋅ ha^−1^ compared to $100.98 ± 3.49 ⋅ ha^−1^ across the CM watermelon fields. The insecticide program for CM watermelons had a CRY of 0.70%; however, all fields within the CM system failed to reach this threshold, and the insecticide applications were never cost-effective. The increased yield from wild pollinator enhancement in the IPM system would result in a financial gain of $4,512.69 ⋅ ha^−1^ over the CM system, based on the previous 5-y regional sale price for seedless watermelon (52).

## Discussion

IPM-based approaches, ones that prioritize treating only when insect pests are present at damaging levels, have become increasingly rare across a range of commodities. Instead, a suite of prophylactic approaches to pest management—including insecticidal seed treatments, soil drenches, and calendar sprays—now dominate most US cropping systems, including the corn and watermelon systems studied here. However, our comprehensive field experiment demonstrates that there is no clear rationale supporting this approach from multiple perspectives including insect pest damage and abundance, pollinator visitation and efficiency, environmental pesticide residues, or crop yield and profitability. These varied and integrative perspectives are vital for grower adoption but surprisingly rare in practice. Hundreds of studies, for example, have tested the negative effects of neonicotinoids and related insecticides on pollinator health in the laboratory and field. The potential threat from these products is incontrovertible. Yet pollination alone paints an incomplete picture without corresponding data on pest population dynamics and crop production. In previous studies that experimentally reduce insecticide use in crops to determine impact on pollinators, the implications for pests and crops are typically overlooked or omitted [e.g., canola (54), cucurbits (49, 55), apples (56), and sunflowers (57)]. Similarly, in studies in which landscape complexity is used as a predictor of pollination services (58, 59), wholesale changes in pest management practices are not explicitly measured or discussed. Farmers are unlikely to change their management practices—no matter how detrimental to bees—if foregoing insecticide treatments leads to excessive crop and economic damage. Conversely, studies on pest/yield relationships [with limited exceptions (60, 61)] involve self- or wind-pollinated crops (7, 11, 62). These experiments often fail to capture the additional losses to yield that nearby or adjacent crops could experience—even though, in some cases, the landowner/crop producer is the same individual.

### Insecticide Use, Pest Outbreaks, and Crop Yield.

One expected corollary of reducing insecticide inputs over years of the experiment was an increase in pest densities over time. Surprisingly, the only evidence of increasing pest pressure on untreated corn was higher damage from rootworm larval feeding in year 4. To isolate the effect of NSTs with minimal confounding factors, corn in our experiment was grown somewhat atypically: without any Bt traits or crop rotation. Therefore, IPM corn was cultivated under a “worst-case scenario” with no protection for the duration of the study. Despite being entirely defenseless for four consecutive years, only three of the five fields experienced increased root feeding and only in the final year. These locations were at the northernmost sites, which is the region of the state, where rootworm pressure is historically highest (63). This outcome demonstrates that corn rootworm populations in major production areas should not be left unchecked and can increase in a relatively short time but that the industry standard of Bt corn with soybean rotation likely maintains rootworm at sufficiently low levels. It is also important to note that, while we focus on rootworm as the primary corn pest, and one for which we observed some evidence of feeding damage, NSTs are largely marketed as targeting secondary pests (e.g., wireworm and seedcorn maggot). These taxa were not present at appreciable densities in any of our experimental fields. Although these cryptic belowground insects are hard to directly sample, indirect evidence of their presence and impact (e.g., poor plant stand in early-season corn) was never observed.

Despite the rise in rootworm damage over time in NST-free corn, yields were not significantly different across the two systems, reinforcing other published studies that show no yield benefit from NSTs (8, 11, 14). Interestingly, the only factor impacting corn yield had nothing to do with insecticide use. We observed gradual but consistent reductions over time with year 4 yields 28% lower than year 1 yields. This effect was apparent across both IPM/CM treatments. The outcome is not surprising, as numerous studies have documented that single-species cultivation has negative feedbacks on crop productivity, including corn (64). These data strongly point to crop rotation as a factor in maintaining high corn yields and likely far more critical in mitigating rootworm damage than NST use (12). For the purposes of this study, we more narrowly defined IPM in the context of insecticide use, but a “true” IPM system would employ crop rotation rather than continuous cropping.

Unlike corn, the key insect pest in IPM watermelon colonized in the initial year and was present at moderate densities throughout the entire experimental period, but, similar to the corn system, these elevated densities did not translate to yield reductions, even using the fairly liberal threshold of five beetles per plant. These data suggest that watermelon should be routinely scouted to protect against the rare site or year in which pests, like cucumber beetles, exceed their threshold but can mostly be cultivated without insecticide use (65–68). Notably, we only observed outbreaks of secondary pests—aphids and mites—in the CM system, in which we repeatedly treated the crop with insecticides. Cucurbit growers in our region frequently mention these as pests of concern; however, many of these same producers also use repeated applications of pyrethroids and neonicotinoids (69), compounds that are highly disruptive to beneficial insect communities that suppress aphid and mite populations (70). Altogether, these observations imply that overly aggressive treatment with broad-spectrum insecticides trigger secondary pest outbreaks in watermelon and that adopting a scouting-based IPM program with fewer inputs prevents the problem.

A major challenge to scouting adoption is that the CRY for watermelon is <1%, reflecting the reality that insecticides such as pyrethroids are inexpensive relative to other farm inputs (e.g., labor). Moreover, our CRY calculations do not account for the additional cost of scouting in IPM systems, which can be challenging to estimate (69). Some growers scout their own fields for pests, while others hire crop consultants. Similarly, scouting a subset of fields or sporadically observing a few edge plants (versus walking transects with a specified sample number and location) will undoubtedly reduce costs but also accuracy. In our experiment, insecticide costs were ca. $101 ⋅ ha^−1^ in CM compared with $3 ⋅ ha^−1^ in IPM. Thus, scouting would need to add at least $98 ⋅ ha^−1^ to offset the difference. Other factors that affect the reliability of this estimate include the additional cost (e.g., fuel, equipment, and labor) of repeated insecticide applications in CM fields and variation in insecticide price or efficacy. Despite these complexities, Ternest et al. (69) found that the cost of seasonal pest scouting ranges from $29 to $120 for a field, well within our estimated price point for a commercial watermelon grower to see a positive return from scouting.

The economics of scouting and IPM as a whole also vary widely across cropping systems. We primarily consider watermelon for which crop value is relatively high, fields are relatively small, and the pests are mostly aboveground and can be controlled with insecticide sprays. In large acreage row crops such as corn with belowground pests that are both hard to sample and lacking immediate rescue-treatment options, the cost/benefit ratio of scouting may be less favorable. Even among specialty crops, we expect the net value of IPM to be highly variable. Watermelon exhibits a few features that could tip the balance in favor of IPM. Compared with other cucurbits, for example, watermelon has a much higher pest threshold due to its natural resistance to the SCB-transmitted bacterial wilt (*Erwinia tracheiphila*) that kills infected plants (71). Also, seedless watermelon has among the highest reliance on bee pollination (72) and, consequently, the risk of insecticide overuse disrupting fruit production is correspondingly greater in this system. Specialty crops with lower pest tolerances and pollination requirements or those produced in regions with higher pest pressures will experience vastly different trade-offs. These relationships are also dynamic and need to be reevaluated regularly over time. In our region and many other parts of the world, insect invasions [e.g., brown marmorated stink bug (73), spotted winged drosophila (74), and spotted lanternfly (75)] result in a constantly changing landscape of pests and the economics underlying their management.

### Routes of Insecticide Exposure for Pollinators.

Neonicotinoids were consistently found at higher levels in the pollen of both crops within the CM system compared to IPM. The specific concentrations detected are comparable with related studies. For instance, squash pollen contained 15 to 19 ng/g of imidacloprid 7 wk postapplication (76) compared to a median value of 6.28 ng/g in this experiment. A trial across the cantaloupe flowering period ranged from 3 to 141 ng/g imidacloprid (77), demonstrating the wide range of potential exposure. Some of this variation is likely explained by bloom time, as we documented much higher levels in early than late flowers. This temporal effect is not trivial. Growers receive price premiums for early melons, and these data indicate that the most valued early flush of flowers are the ones that are most heavily contaminated with neonicotinoids.

Bees were also likely exposed via soil residues. Recent studies emphasize the significance of soil-derived neonicotinoid exposure for ground-nesting bees, including imidacloprid in cucurbits (48, 49, 55). This difference in exposure could partly explain why we observed treatment effects on floral visitation for wild bees (most of which are ground nesters) and not managed honey bees. However, this differential response among pollinators is likely driven in part by other factors inherent to honey bee biology and management (e.g., hives are stocked at high densities, with >20,000 individuals per colony; large individual body size, and thus pesticide tolerance, compared to many solitary wild species). A recent field experiment on commercial cucurbit farms in the midwestern United States similarly found that insecticide use reduces wild bee visitation with no corresponding effect on honey bees (78). This effect is notable, since wild bees in our experiment were both most sensitive to insecticide use and most strongly correlated with crop yield. The latter outcome should be expected—wild bees, in general, are more efficient than honey bees as crop pollinators (79–81), and in watermelon, wild bees are more than twice as effective on a per-capita basis in promoting fruit set and growth (81, 82).

A limitation of our experimental design is that we are unable to differentiate the relative influence of corn and watermelon inputs on crop pollination, since the two are confounded (i.e., we did not independently manipulate insecticide use across the two crops in a factorial design). Because the crops were treated with different neonicotinoids—thiamethoxam in corn and imidacloprid in watermelon—we can infer mobility and exposure across these crop types by interpreting residues from these active ingredients. Clothianidin, for example, was detected at low levels in 72% of CM watermelon pollen in 2019 compared to 0% in IPM pollen despite never being applied to watermelon in either treatment. These patterns suggest that watermelon roots scavenge these compounds from a pool of soil residues derived from either ground water movement from the surrounding corn or carryover effects due to the prior year’s NST corn planting. Another likely possibility is that highly mobile bees foraged across crop boundaries, which were well within the flight radius of most taxa. Generalist pollinators like bumble bees tend to avoid cucurbit pollen (83) and readily forage on corn pollen when little else is available (84). Indeed, we observed few bumble bees foraging on watermelon flowers (<10% of visits; *SI Appendix*, Fig. S5) despite stocking fields with managed hives. However, more information is needed on the foraging ranges and behaviors of nonhoney bee taxa across crop boundaries; for example, the longhorn bee *Melissodes bimaculatus* is an extremely common, mobile, and effective wild pollinator, but its movement within or between crop fields is poorly documented.

A final outcome worth emphasizing is the speed with which the pollinator community responded to IPM implementation. Improvements to bee visitation and yield were observable rapidly, in the first year of the experiment (Fig. 2), even though these farm sites were conventionally managed in previous years and surrounded by conventional agriculture. The response did not require multiple years of insecticide reduction or installation of pollinator habitat. There is a perception that farmland in its current state is devoid of natural life, but these data show that reduced inputs alone, independent of habitat or land use changes, can have demonstrably positive effects in the near-term.

## Conclusion

One of the central challenges of global food security in the 21st century is ensuring adequate food supply for a growing population while conserving natural resources. These are often viewed as contradictory endeavors (i.e., a trade-off between agricultural productivity and conservation). Indeed, “feeding the world” is a common rationale for excessive pesticide use and insurance-based pest management approaches in crop protection. Yet, increasingly, studies find that substantially lower pesticide inputs result in equivalent yields (85), suggesting that high productivity can be maintained—or even increased, as shown in our study—with less intensive management. This finding dovetails the recent call for ecological intensification of agriculture, for which IPM adoption is a central theme (86–88).

Overall, our study demonstrates that the current, prophylactic approaches offer no consistent benefits to offset the demonstrably negative impacts to both pollinators/pollination and crop yields. The convenience of NST and calendar sprays to manage pests is clearly attractive to some producers. However, this argument rests on the twin assumptions that 1) populations of target pests can be expected to be at economically damaging populations each year, and 2) monitoring-based IPM alternatives expose producers to higher risks and/or upfront costs. Our data do not offer support for these claims in either cropping system and, in fact, show that embracing the use of IPM may offer a readily available “win-win” scenario for crop production and pollinator health across diverse crops.

It is important to note that conducting pest surveys with economic thresholds is not a new phenomenon; thus, our approach was not revolutionary and did not reinvent the wheel. The tools, in principle, have been established for decades, even if they have fallen out of practice. A key step forward is better understanding the thought process underlying when and why farmers decide to use insecticides. There is a myth that farmers only care about profit and refuse to monitor pests because it is too much effort or too time-consuming. Neither of these seem to be universally true. In a recent grower survey of reasons for implementing action thresholds, saving money on insecticide sprays was not among the top three responses and ranked beneath “less harmful to the environment” (89). Similarly, “reducing scouting” and “convenience” were among the bottom several reasons when soybean farmers were surveyed about their pest management decisions in the context of seed treatments, whereas “protecting water quality” and “public safety” were among the top factors (90). These trends are validated by the success of previous extension-based programs in helping growers adopt IPM tactics (89). However, IPM adoption has a long and rocky history that extends far beyond grower education efforts (91–95). This circumstance is particularly complicated for seed treatments in which growers may not be making explicit decisions to use neonicotinoids, since they are typically the default option offered by seed suppliers (16). In this case, an “extended peer community” that engages farmers, consumers, industry, government, and conservation programs will be vital (96) while ensuring that choice is maintained in crop seed sales and that growers are provided with clear guidelines for how to implement scouting using scientifically backed pest thresholds.

## Materials and Methods

### Site and Experimental Design.

The experiment was conducted over 4 y (2017 to 2020) on five research farms at the Purdue Agricultural Centers (PACs) located across Indiana (*SI Appendix*, Fig. S1): Northeast (NEPAC; Columbia City, IN), Pinney (PPAC; Wanatah, IN), Throckmorton (TPAC; Lafayette, IN), Southeast (SEPAC; Butlerville, IN), and Southwest (SWPAC; Vincennes, IN). These sites are positioned along a latitudinal gradient across the state with at least 100 km separating one another, ensuring that sites represent a diversity of climatic conditions, soil types, and local pest pressures.

Each site contained of a pair of agricultural fields that were randomly assigned to either a CM or IPM program. These treatments were designated in year 1 of the study (2017) and remained within this management system for the duration of the experiment. CM systems were considered the “industry standard” and were designed to mimic the pest management regime typically found in both row crops and vegetable production, including the routine use of prophylactic insecticides. The IPM system was an experimental treatment that relied on pest scouting to determine the use of insecticides. We only applied insecticides as needed based on published action thresholds as specified in *SI Appendix*, *Supplemental Methods*. Within a site, paired fields were separated by an average of 5.6 km (range: 4.63 to 6.63 km), which resulted in similar abiotic conditions (e.g., temperature and precipitation) while providing sufficient buffer for biological independence of CM/IPM treatments, as insect pollinators are unlikely to fly >5 km (97).

### Cropping Systems.

Fields (area mean: 5.74 ha, range: 4.82 to 7.73 ha) were planted continuously with corn in all 4 y of the study. While corn–soybean rotation is common in the midwestern United States (72.3% of all corn acreage in key corn producing states—Iowa, Illinois, and Indiana—from 2015 to 2019), continuous corn is the next most prevalent system, constituting 24.7% of acres (52). Starting in year 2 of the study (2018) and continuing for three growing seasons, we planted a 0.2-ha watermelon plot embedded centrally within the corn matrix (*SI Appendix*, Fig. S2). Corn is the dominant crop grown in Indiana and throughout much of the Midwest (11.74 million ha across Iowa, Illinois, and Indiana). Thus, this design is a microcosm of midwestern US agriculture, in which pollinator-dependent crops such as watermelon are bordered, and often completely surrounded, by corn. The goal of this design was to document the effects of large field crop plantings upon other, adjacent cropping systems. Corn was planted 1 y in advance of watermelon because neonicotinoid exposure can occur both in season through a variety of exposure routes or from the previous year’s inputs. This aspect of the experimental design reflects that the vast majority of watermelon acreage on Indiana farmland (77%) is in rotation with either corn or soybean (52). Management practices (e.g., tillage, irrigation, fertilizer, herbicides, and fungicides) were standardized across sites such that the only factors differentiating CM/IPM field pairs were insecticide inputs (refer to *SI Appendix*, *Supplemental Methods* for management details and field histories).

All corn seed (Spectrum 6334) across both treatments received a fungicide seed treatment (Maxim Quattro: azoxystrobin 2.5 µg; fludioxonil 6.5 µg; mefenoxam 5 µg; thiabendazole 50 µg active ingredient [a.i.] ⋅ seed^−1^); however, CM corn seed was also treated with the neonicotinoid thiamethoxam applied at the maximum rate, marketed for control of corn rootworms and a suite of other secondary pests (Cruiser 5FS at 1.25 mg a.i. ⋅ seed^−1^). By 2012, >80% of all US corn seed was coated with at least one neonicotinoid (15), and the CM treatment thus represents the corn seed most commonly used by US farmers. Throughout the experiment and in both treatments, we used a nontransgenic variety that did not express Bt toxins (*Bacillus thuringiensis*), meaning that the untreated IPM seed was unprotected from larvae of the western corn rootworm (*D. virgifera virgifera* LeConte), the key corn insect pest in the region, and other soil insect pests. This allowed for a “true” assessment of the efficacy of NST impacts on pest control without the confounding effects of multiple, layered plant protection technologies. However, in practice, all corn seed sold in the United States that expresses Bt toxins is also treated with at least one neonicotinoid insecticide (98).

We used a seedless watermelon system, which requires triploid and diploid plants interspersed with one another. All watermelon fields contained the triploid variety ‘Fascination’ as the seedless crop along with the diploid var. SP-7 as the pollenizer at a 3:1 ratio to ensure adequate pollination. At transplant, CM watermelons were treated with the neonicotinoid imidacloprid (Wrangler at 814.09 mL/ha) as a soil drench at the high rate, while IPM watermelons received no insecticides. Additionally, CM watermelons were sprayed with the high rate of the insecticide lambda-cyhalothrin (Warrior II pyrethroid at 140.3 mL/ha) via tractor-drawn air blaster or boom sprayer at 4, 6, 8, and 10 wk posttransplant, resulting in four foliar applications each season. Application rates for both insecticides (standardized by milliliter a.i. per hectare; lambda-cyhalothrin = 31.98, imidacloprid = 316.43) are within the range recommended by the label (lambda-cyhalothrin = 21.32 to 31.98, imidacloprid = 237.94 to 356.91). Similarly, insecticide rates used in the experiment are slightly higher than, but comparable to, those applied by watermelon growers in our region, according to on-farm pesticide records reported in ref. 69: lambda-cyhalothrin (*n* = 18 applications; mean = 26.93, median = 26.66, range = 16.66 to 33.32), imidacloprid (*n* = 7 applications; mean = 293.92, median = 297.43, range = 250.22 to 328.41).

Although watermelon insecticide regimes across growers are more diverse than corn, our prior on-farm survey of insecticide use on 17 Indiana watermelon farms found that producers averaged ∼5 treatments per field per season, and thus the five applications in the CM treatment (1 soil drench + 4 foliar sprays) were intended to reflect this practice (69). The survey further revealed that pyrethroids, including lambda-cyhalothrin, were the three most used active ingredients. Neonicotinoids, including imidacloprid, were also used but at lower frequencies (30% of watermelon growers in ref. 69). These data guided our pyrethroid-biased regime in the CM treatment. Watermelons in the IPM treatment were left untreated unless insect pests exceeded economic thresholds at a site (see *Insect Pest Abundance and Damage*), in which case the field was also treated with a foliar spray of lambda-cyhalothrin, as described for CM fields. Additional details on corn and watermelon management (e.g., planting dates and seeding rates) are provided in *SI Appendix*, *Supplemental Methods*.

The watermelon–corn matrix was supplemented with managed bees to replicate the pollination practices used by commercial watermelon growers, who typically either rent honey bee hives from beekeepers or purchase bumble bee hives. Increasingly, growers in our region stock with both honey bees and bumble bees in the same field due to their foraging at different times and weather conditions. In each field, two honey bee colonies were placed on opposite corners at the edge of watermelon plots in an arrangement that avoided interference with pesticide application. This stocking rate (1 hive per 0.1 ha) falls within the recommended range for commercial production used by regional growers (99). Additionally, one Quad pollination hive (Koppert Biological Systems) containing four bumble bee (*Bombus impatiens*) colonies was placed in each field at 4 to 5 wk posttransplant to synchronize activity with the watermelon bloom period.

### Insect Pest Abundance and Damage.

Corn plants were evaluated for both early- and late-season pest damage to assess the efficacy of insecticidal seed treatments. Because foliar insect pests were rarely observed, sampling focused on the more economically damaging guild of soil-dwelling root pests. First, corn stand was evaluated at the V3 to V4 stage, along six 5.3-m transects down a row, in which the number of emerged plants was counted. Transect counts were averaged and extrapolated to estimate plants per hectare and compare with known planting densities. Poor corn stand, relative to initial planting rates, is often an indication of belowground seedling damage by insects including wireworms and seedcorn maggots (100, 101). At corn anthesis, root damage was quantified to determine potential for lodging due to corn rootworm feeding. In every field, 10 random plants were excavated along each of four transects that were >20 rows from the field edge with >10 m separating sampled plants within a transect. The root mass was then rinsed and evaluated for damage using the Oleson injury rating scale (102), the established approach for assessing rootworm feeding.

Beginning the week following transplant, watermelon plants were surveyed for pests weekly for a 10-wk period extending to harvest. Each survey consisted of five randomly positioned transects, with plants sampled at 10, 20, and 30 m from the plot edge (*n* = 15 plants per plot per week). For each plant, all aboveground tissue was inspected, and the identity and number of insect pests found on the plant or the soil directly below were recorded. If the density of the primary pest, SCB *A. vittatum* (F.), exceeded the economic threshold of five adult beetles/plant, then the plot was treated with a foliar spray of lambda-cyhalothrin within 2 d of the observation (103). Refer to *SI Appendix*, *Supplemental Methods* for additional details on pest scouting protocol.

### Watermelon Pollinators.

To assess pest management impacts on pollination, we conducted visual observations of watermelon flowers to quantify pollinator visits and community composition. Flower clusters, consisting of at least five male and one female flower, were observed for a 3-min period, during which pollinator type, number of flowers visited, and transition of pollen from a male to female flower (i.e., a pollination event) were recorded. Behavioral observations were conducted on the same date at both fields at each site. First observation began 5 to 6 wk posttransplanting and continued for 5 consecutive weeks to encompass most of the blooming period that contributes to harvested yield. Refer to *SI Appendix*, *Supplemental Methods* for more detail on sampling design.

### Crop Yield.

Corn maturity was monitored, and the crop was harvested during each of the 4 y to assess the impact of NSTs on yield. All yield reports were adjusted to account for variation in moisture at harvest, and data were standardized to a 15.5% moisture content.

Because corn yields were strongly affected by local factors (e.g., soil type, pH, and drainage) determined by random field assignment, we conducted a separate companion study in 2019 using the same two corn seed treatments. This higher-resolution study focused exclusively on yield in smaller, more highly replicated plots with both treatments (neonicotinoid-treated versus untreated) included in the same field to control for site variation. The trial was repeated at six sites; four of the five original PACs used in the experiment (all but SEPAC) and two additional locations (Davis PAC in Farmland, IN, and the Agronomy Center in West Lafayette, IN). At each site, we planted four to nine replicates of two adjacent 5.3-m-length rows of each corn treatment in a randomized complete block design with the same planting date across all replicates at each site (*n* = 33 total plot replicates for both treated and untreated seed). At harvest, the weight and moisture adjusted yield for each replicate was extrapolated to a per-hectare yield.

Beginning at fruit maturity (approximately 80 d), five randomly positioned subplots (5 × 2 m area) of each watermelon field were hand-harvested and used to estimate yield. Mature fruits from each subplot were counted, weighed, and inspected for marketability using US Department of Agriculture (USDA) grading standards (104) for lack of physical deformities or disease. Subplots were harvested weekly for four consecutive weeks, after which data were summed over time to calculate a total yield per unit area.

### Pest Management Profitability.

Cost of insecticides applied were either calculated from direct expenditures from purchased product or sourced from external guides (105). The cost of the product (Cruiser 5FS) applied as an NST could be quantified but fails to account for additional costs of seed treatment practices that include labor, infrastructure, specialized equipment, and transportation. A proxy for this calculation can be used based on industry-provided costs for the other commonly used neonicotinoid in corn pest management, clothianidin (53). We also calculated the CRY, which is interpreted as the minimum percentage in yield gain required to cover the cost associated with an insecticide treatment and reach a breakeven point at which the treatment cost is recuperated (6, 106, 107). CRY was calculated by dividing the insecticide treatment cost by the crop price × crop yield. For both watermelon and corn, price and yield were based on the previous 5-y average (2016 to 2020) from the state of Indiana (52).

### Pesticide Residues.

Samples of soil, watermelon leaf tissue, and corn and watermelon pollen were collected during each of the 4 y and analyzed to detect residues of insecticides and fungicides applied to both corn and watermelon crops using the QuEChERS procedure, followed by liquid chromatography–tandem mass spectrometry (LC-MS/MS) for pesticide identification and quantification. Refer to *SI Appendix*, *Supplemental Methods* for sample number, preparation, and analytical details.

### Statistical Analysis.

All statistical analyses were performed using SYSTAT 13 (SYSTAT Software, Inc) by creating a series of general (continuous data) or generalized (discrete data) linear models. To avoid pseudoreplication, all data points were condensed to a single year/site/treatment to be used in the model by taking the mean for damage evaluations across dates and yield measurements within a field as well as summing pest counts or pollinator measurements across observation dates for each field. This process resulted in 40 and 30 data points for corn and watermelon, respectively, per response variable; crop differences were due to corn being cultivated for one extra year (2017) than watermelon (*Cropping Systems*). Stand counts were natural log–transformed, while root damage at each site was summed and multiplied by 100 to produce integer values and then fit to a zero-inflated distribution. SCB counts and pollinator surveys were summed as total number of beetles or pollinators at each field, to maintain discrete integer values, and fit with a negative binomial distribution. Corn and watermelon yield data were normally distributed and remained untransformed. Models used year (*n* = 4 corn, *n* = 3 watermelon), site (*n* = 5), and management treatment (*n* = 2) as fixed effects as well as two-way interactions between treatment and year or site. Post hoc pairwise comparisons (Fisher’s least significant difference) were used to differentiate any factors (or interactions) that were significant. Within-field corn yield assessment was analyzed in a separate mixed model with the use of NST and site (*n* = 6) as fixed effects and spatial block as a random effect. The relationship between crop yield and pollinator visits was explored with regression analysis with a fixed effect of treatment. This relationship was tested against the number of visits from honey bees and the wild pollinator community to contrast the effect from managed versus wild pollinators. Raw data generated from this study are publicly accessible in the Purdue University Research Repository (109).

## Data Availability

Raw data have been deposited in the Purdue University Research Repository (DOI: 10.4231/4DQF-3G13).
